# Flaxseed and Carbohydrase Enzyme Supplementation Alters Hepatic n-3 Polyunsaturated Fatty Acid Molecular Species and Expression of Genes Associated with Lipid Metabolism in Broiler Chickens

**DOI:** 10.3390/vetsci6010025

**Published:** 2019-03-08

**Authors:** Brian Head, Massimo Bionaz, Gita Cherian

**Affiliations:** 1 Linus Pauling Institute, Oregon State University, Corvallis, Oregon, OR 97331, USA; Brian.Head@oregonstate.edu; 2 Department of Animal and Rangeland Sciences, Oregon State University, Corvallis, OR 97331, USA; Massimo.Bionaz@oregonstate.edu

**Keywords:** broiler chicken, flaxseed, n-3 fatty acid, phospholipid species, gene expression

## Abstract

Flaxseed is rich in α-linolenic acid and is used in broiler chicken diets to enrich tissues with n-3 fatty acids (FA). However, non-starch polysaccharides (NSP) in flaxseed decreases nutrient digestibility and limits the availability of n-3 FA. Addition of carbohydrase enzymes to flaxseed-based diets can decrease the anti-nutritive effects of NSP. We hypothesized that flaxseed and enzyme supplementation affect lipid content and alter expression of genes related to lipid metabolism in broiler liver. Five day-old broiler chicks were fed a corn-soybean basal diet with 0% flaxseed, a basal diet with 10% of flaxseed, or 10% flaxseed + 0.05% enzyme diet up to day 42 of growth. Total lipids, including long-chain (≥20C) n-3 FA and monounsaturated FA, were increased in flax-fed broiler livers. Enzyme addition reduced arachidonic acid and total long chain n-6 FA. These changes were similarly reflected in phosphatidylcholine lipid species. Dietary flax and enzyme treatments up-regulated PPARα target genes *CPT1A* and *ACOX1* while reducing expression of *de novo* FA synthesis-related genes. This study concludes that flaxseed and enzyme supplementation in broiler diets enhances LC n-3 FA species, while reducing n-6 FA species in hepatic phospholipids (PL). Flaxseed-based diets changes the expression of genes involved in FA lipid metabolism without affecting growth or production performance in broilers.

## 1. Introduction

Long chain (LC) (≥20C) polyunsaturated fatty acids (PUFA) such as arachidonic acid (ARA, 20:4n-6), eicosapentaenoic acid, (EPA, 20:5n-3), and docosahexaenoic acid (DHA, 22:6n-3) are derived from dietary essential n-6 linoleic acid (18:2n-6, LA) and n-3α-linolenic acid (18:3n-3, ALA). Dietary fatty acids (FA) provided to avian species are trafficked to the liver, which acts as a lipid sink and the primary site of *de novo* lipogenesis. In the liver, synthesized LC PUFA go on to form critical membrane structural components and provide necessary physiological roles in the vertebrate body [1]. PUFA, stored primarily in membrane phospholipids (PL), are significant modulators of metabolism, driving biochemical shifts to produce and catabolize an array of second messenger metabolites [2]. Phosphatidylcholine (PC) and phosphatidylethanolamine (PE) constitute the greatest fraction of PL that maintain significant unsaturation indices relative to other lipid reservoirs, such as the highly saturated triglycerides (TAG). LC PUFA stored in PC and PE contribute to membrane fluidity and biogenesis [3]. Phospholipid FA composition is highly associated with dietary n-3:n-6 FA ratios. Thus, ALA from the diet, by itself or as a precursor to EPA and DHA, may then be incorporated into membrane lipids to further alter membrane dynamics, second messaging capacity and metabolism [2,4].

Lipogenesis in the broiler chicken liver is altered by dietary interventions [5]. As a hub of exogenous fat supply and lipid synthesis, the liver integrates metabolic signals through several lipid-metabolism related transcription factors and protein coding genes. Sterol regulatory-element binding proteins (SREBP) are a family of transcription factors associated with nutritional homeostasis, specifically regulating hepatic and whole-body FA, TAG and cholesterol synthesis [6]. Sterol regulatory-element binding factor 1 (SREBP-1) promotes the transcription of key lipogenic genes, such as fatty acid synthetase (*FASN*) and acetyl-CoA carboxylase α (*ACACA*) in addition to genes coding for proteins necessary to produce monounsaturated FA (MUFA) and PUFAs, such as the desaturase fatty acid desaturase 1 and 2 (*FADS1*, *FADS2*) and stearoyl-CoA desaturase 1 (*SCD1*) and various elongases such as *ELOVL2*, *ELVOL5*, and *ELOVL6* [6,7,8]. Contrasting with lipogenesis is the FA catabolic pathway regulated by the peroxisome proliferator-activated receptor (PPAR) family of nuclear receptors. PPARα, the predominant hepatic isoform, instigates FA acyl chain activating pathways ultimately to alter FA trafficking toward β-oxidation [8]. Downstream targets of PPARα activation include carnitine palmitoyl transferase 1a (*CPT1A*), acyl-CoA oxidase 1 (*ACOX1*), microsomal triglyceride transfer protein (MTTP) and fatty acid binding protein 1 (FABP1) [9]. Both SREBP and PPAR are intrinsically associated with nutritional homeostasis and have established interactions with intracellular lipids, and specifically n-3 PUFA [10]. Our hypothesis is that dietary ALA from flaxseed are further elongated, desaturated and enrich hepatic PL molecular species and alter expression of genes related to lipid metabolism in broiler liver.

ALA-rich flax oil and whole flaxseed has been established as a dietary supplement to enrich poultry meat with n-3 FA [11,12]. Although a rich source of ALA, whole flaxseeds contains a variety of anti-nutritional factors associated with reduced overall nutrient digestibility, including soluble and insoluble non-starch polysaccharides, mucilage and cyanogenic glycosides [13]. To increase flaxseed digestibility in the present work we utilized carbohydrase enzymes, which prior research has shown to increase ALA and n-3 FA incorporation in broilers fed flaxseed diets [14,15]. The objective of our work was to evaluate the effect of adding commercially available carbohydrase enzymes in combination with flaxseed supplementation on hepatic lipid content, PL molecular species composition, and lipid metabolism-related gene expression profiles in broiler birds.

## 2. Materials and Methods

### 2.1. Animal Care and Diets

An institutional animal care and use committee approved all experimental protocols to ensure adherence to animal care guidelines. A total of twenty-four, five-day-old broiler chicks (White Cornish Cross Straight Run) were weighed individually, distributed individually in cages (53 cm × 48 cm × 46 cm, length × width × height) and were randomly allocated to three treatment groups. The chicks were fed a corn-soybean basal diet with 0% flax seed (control), or a control diet with 10% of flaxseed (wt/wt) (*Linum usitatissimum* L.) (flax) or 10% flaxseed + enzyme (flax+E) (Table 1) from day five through day 42 of growth. The Omegazyme carbohydrase enzyme mixture (Canadian Bio-Systems, Calgary, AB, Canada) was provided at 0.05 g/100 g prepared feed in the flax+E treatment. The enzyme mixture included cellulase (2800 U/g), xylanase (1000 U/g), glucanase (600 (U/g), mannanase (400 U/g), galactanase (50 U/g), amylase (1000 U/g) and protease (200 U/g). Chicks were kept in 23 h light and 1 h dark for the entire growing period. Water and feed were provided ad libitum. Each cage is considered as the experimental unit. The chicks did not receive any vaccines or drugs during the entire experimental period. Chicks were individually weighed on day 5, 22 and day 42 of growth. At the end of feeding trial, chickens were euthanized by CO_2_ inhalation, decapitated, and were cut open, liver tissue was removed, washed with saline, weighed and kept frozen at −80 °C until analysis. A portion of liver tissue was removed, flash frozen in liquid nitrogen and stored at −80 °C for RNA extraction. The muscle tissues (right pectoralis major and right biceps femoris) were dissected and weighed.

### 2.2. Lipid Profiling

Total lipids were extracted from approximately 2 g of feed or liver tissue samples using a 2:1 solution of chloroform and methanol [16]. Fatty acid methyl esters were prepared with boron trifluoride methanol as the methylating agent using methods reported earlier [15]. Fatty acid analysis was performed with a HP 6890 gas chromatograph (Hewlett-Packard Co., Wilmington, DE, USA) equipped with an autosampler, flame ionization detector, and SP-2330 fused silica capillary column (30 m × 0.25 mm × 0.2 μm film thickness) (Supelco, Bellefonte, PA, USA). Samples in hexane (1 μL) were injected with helium as a carrier gas into the column programmed for ramped oven temperatures. Initial oven temperature was set at 150 °C, held for 1.5 min, then ramped at 15 °C/min to 190 °C and held for 20 min, then ramped again at 30 °C/min to 230 °C and held for 3 min. Inlet and detector temperatures were both 250 °C. Fatty acid methyl esters were identified by comparison with retention times of authentic standards (Catalog no:1177, Matreya LLC, State College, PA, USA). Peak areas and percentages were calculated using Hewlett-Packard ChemStation software (Agilent Technologies Inc., Wilmington, DE, USA). Fatty acid values are reported as mg/g tissue and g/100 g (%) of FA methyl esters.

### 2.3. Analysis of Phospholipid Molecular Species

Polar lipid classes and molecular species in the liver tissue was determined by direct infusion electrospray ionization (ESI) and tandem mass spectrometry (MS/MS) at the Kansas Research Center analytical laboratory. An automated ESIMS/MS approach was used, and data acquisition, analysis and acyl group identification were carried out, with some modifications as listed on the Kansas Lipidomics Research Center website [17] and as per our previous research [18]. Peak intensities were used for relative quantification of fatty acid species in broiler liver tissue and displayed as response.

### 2.4. RT-qPCR of Lipid Metabolism-Related Genes

Hepatic tissue was weighed (~0.1 g) and immediately subjected to RNA extraction using ice-cold Trizol (Invitrogen Corp., Carlsbad, CA, USA) as described by Rosa et al. [8]. Genomic DNA was removed from RNA with DNase using Direct-zol RNA MiniPrep Kit (Zymo Research Corp., Irvine, CA, USA). RNA concentration was measured using a NanoDrop ND-1000 spectrophotometer (www.nanodrop.com). The purity of RNA (A260/A280) was 1.86 ± 0.16 (mean ± SD). RNA integrity was assessed by 2100 Bioanalyzer Instrument (Agilent, Santa Clara, CA, USA) by the Center for Genome Research and Biocomputing at Oregon State University. The RNA integrity number was 7.8 ± 1.0.

cDNA was synthesized using 100 ng RNA, 1 ug dT18 (Invitrogen Corp.), 1 μL 50 μM random primers (Invitrogen Corp.), and 11.625 μL DNase/RNase free water. The mixture was incubated at 65 °C for 5 min and kept on ice for 3 min. A master mix composed of 4 μL 5× First-Strand Buffer (Thermo Scientific, Waltham, MA, USA), 0.25 μL RevertAid Reverse Transcriptase (Thermo Scientific), 1 μL 10 mM dNTP mix (Invitrogen Corp.), and 0.125 μL RiboLock RNase Inhibitor (Thermo Scientific) for each 20 μL cDNA reaction. The reaction was performed in a Veriti 96 Well Thermal Cycler (Applied Biosystems, Foster City, CA, USA) using the following program: 25 °C for 5 min, 50 °C for 60 min and 70 °C for 15 min.

Quantitative PCR (qPCR) was performed using 4 μL diluted cDNA combined with 6 μL of a mixture composed of 5 μL 1× SYBR Green master mix (Applied Biosystems), 0.4 μL each of 10 μM forward and reverse primers, and 0.2 μL DNase/RNase free water in a MicroAmpR Optical 384-Well Reaction Place (Applied Biosystems). Each sample was run in triplicate plus the non-template control (NTC). The reactions were performed in an ABI Prism 7900 HT SDS instrument (Applied Biosystems) using the following conditions: 2 min at 50 °C, 10 min at 95 °C, 40 cycles of 15 s at 95 °C (denaturation) and 1 min at 60 °C (annealing and extension). The presence of a single PCR product was verified by the dissociation protocol using incremental temperatures to 95 °C for 15 s plus 65 °C for 15 s. Data were captured using the 7900 HT Sequence Detection Systems Software (version 2.2.4, Applied Biosystems) and analyzed using LinRegPCR [19]. The final data were normalized using the geometric mean of four internal control genes, specifically *GAPDH*, *MRPL39*, *MRPS9* and *RPS15A*. Normalization of housekeeping genes had a V-value of 0.097, as assessed by geNORM [20]. Primer features are shown in Tables S1 and S2.

### 2.5. Statistics

The effects of diet on body weight, organ weight, tissue FA, PL FA molecular species and relative gene expression were analyzed by one-way ANOVA with diet as the main factor using the General Liner Model procedure SAS (version 9.4) (SAS Institute, Cary, NC, USA) [21]. Significant differences (*p* < 0.05) among treatment means were separated using Tukey′s HSD test. For 0.05 < *p* < 0.10, results are discussed if means suggested a trend, and a cage was considered the experimental unit. Least square means and pooled standard error of the means (SEM) are reported.

## 3. Results

### 3.1. Diet Lipid Profile

The addition of flaxseed at 10 g/100 g provided experimental diets approximately a 6.7-fold increase of ALA relative to the control (Table 1). Addition of flaxseed led to a reduction in proportion of palmitic acid (16:0) and n-6 linoleic acid (18:2n-6) while increasing the proportion of oleic acid (18:1) and eicosanoic acid (20:1) content in the feed (Table 1).

### 3.2. Chicken Production Performance

No differences were observed between initial body weight or final body weight among birds fed control or flaxseed-based diets. The day 42 body weight of the broiler chickens were 1.68, 1.59 and 1.70 kg for control, flax and flax+E, respectively (*p* > 0.05) (Table S3). The relative yield (expressed as percent of body weight) of commercially relevant organs such as breast muscle and thigh muscle did not differ among the treatment groups (*p* > 0.05) (Table S3). The heart weight of day 42 birds fed flax+E were smaller than both control and flax fed birds (Table S3).

### 3.3. Liver Lipid Profile

**Figure 1:**
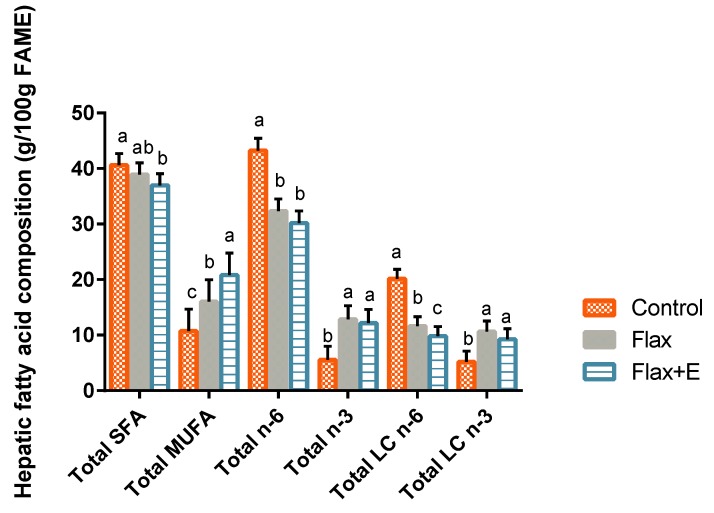
Effect of flaxseed and enzyme supplementation on liver fatty acids. Control, Flax, Flax+E represent corn-soybean meal basal diet (control), and basal diet with 10% whole flaxseed (Flax) plus 0.05% enzyme (Flax+E). The a–c means, with no common superscript within a cluster, differ when *p* < 0.05. *n* = 8. Data is represented as g per 100 g fatty acid methyl esters (FAME). SFA = saturated fatty acids. MUFA = monounsaturated fatty acids. LC = Long chain (>20C).

Amount of total lipids in the liver increased in both flax and flax+E diets (3.59, 3.77 mg/g) compared to the control group (3.31 mg/g) (*p* < 0.05) (Table S4). Hepatic fatty acid profile was significantly affected by the dietary treatments (Figure 1) (Table S5). Livers from flax and flax+E birds were associated with an expectedly reduced proportion of LA and increased proportion of ALA. Long-chain (>18C) n-3 and n-6 FA were also present in the liver tissue with flaxseed-fed birds having lower amounts of ARA and higher amounts of EPA and DHA (*p* < 0.05). Additionally, flax+E liver lipids were markedly lower in total long-chain n-6 FA. Total long-chain n-3 FA, however, were unchanged in flax relative to flax+E livers. The proportion of total saturated FA (SFA) were significantly reduced in flax+E livers substituting for enhanced proportion of total monounsaturated FA (MUFA), relative to control livers. Stearoyl-CoA desaturase index, a ratio of MUFA to SFA in hepatic tissue, was significantly higher in flax+E livers relative to both flax and control livers (Figure S1).

**Figure 2:**
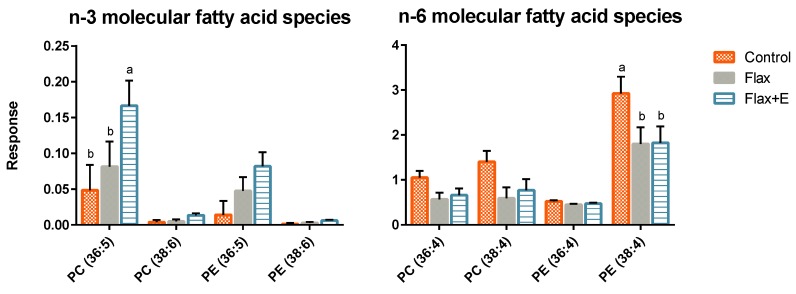
Effect of flaxseed and enzyme supplementation on liver phospholipid molecular fatty acid species. Control, Flax, Flax+E represent corn-soybean meal basal diet (Control), and basal diet with 10% whole flaxseed (Flax) plus 0.05% enzyme (Flax+E). The a–c means with no common superscript within a cluster differ when *p* < 0.05. *n* = 8. Peak intensities were used for relative quantification of fatty acid species in liver tissue.

Phospholipid species in the liver of broilers receiving the different treatments were differentially enriched with FA species displayed as response or peak intensity of identified lipid species (Figure 2). In the lipidomics dataset, PC 36:5 (16:0/20:5) was highest in flax+E livers with a trending increase of PC 38:6 (16:0/22:6), PE 36:5 (16:0/20:5) and PE 38:6 (16:0/22:6) among flax-fed birds. On the contrary, arachidonic acid species (38:4; 16:0/20:4; 18:0/20:4) were significantly decreased in PC and PE of flaxseed-fed birds (Figure 2).

### 3.4. Liver Lipid Metabolism-Related Gene Expression

**Figure 3:**
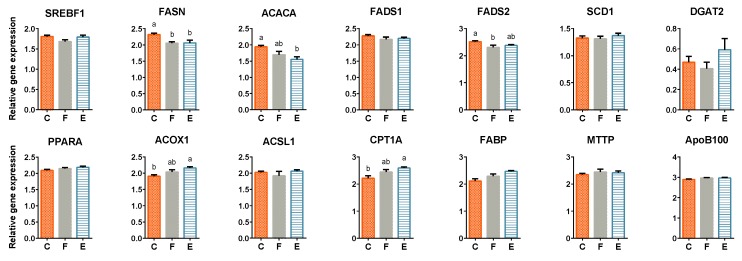
Effect of flaxseed and enzyme supplementation on relative gene expression patterns for select lipid metabolism-related genes in broiler hepatic tissue. Values are log_10_ transformed, least square means ± standard errors of the mean. Labels a–b denote significant difference (*p* < 0.05) among treatments. C, F and E represent corn-soybean meal basal diet (C, Control), and basal diet with 10% whole flaxseed (F, Flax) plus 0.05% enzyme (E, Flax+E).

Hepatic tissue of flax+E broiler chickens had significantly increased expression of genes related to fatty acid catabolism *ACOX1* and *CPT1A* relative to control (Figure 3, Tables S1 and S2). Expression of other measured genes related to transcriptional regulation (*PPARA*), fatty acid uptake, activation, and transport (*FABP1*, *ACSL1,* and *SLC27A2*), triglyceride synthesis (*DGAT2*), and VLDL formation (*APOB* and *MTTP*) was not affected by dietary treatments in the hepatic tissue.

Few of *de novo* lipogenesis-related genes were significantly down-regulated in hepatic tissue of broilers fed whole flaxseed, such as *FASN* and *FADS2*. Flax+E birds had reduced transcription of *FASN* and *ACACA* compared to control birds. No effect of diet on expression of other genes involved in *de novo* synthesis, including *SREBF1*, *FADS1*, *ELOVL6*, and *SCD1* was observed in the liver tissue. Relevant gene expression patterns are displayed in Figure 3. Relative gene expression of *SLC27A2* and *ELOVL6* were statistically unchanged across experimental treatments, not shown graphically.

## 4. Discussion

The current study suggests that a diet comprised of 10% flaxseed by weight with an increased dietary ratio of ALA:LA relative to a standard broiler diet is sufficient to alter lipid content, PUFA profile, phospholipid molecular species and generate significantly different lipid-metabolic gene expression patterns by day 42 in broiler chicken liver tissue. 

Our data indicated that flaxseed-based diet significantly increases total lipids in the liver, the primary site of *de novo* lipid synthesis in the broiler bird. Prior work reported a decrease of liver fat mass upon increase of dietary PUFA in broiler chickens [22]. In our experiment the liver fat mass was instead increased by dietary PUFA, albeit different dietary composition and in a n-3:n-6 ratio. The more abundant hepatic lipids may be a response to, or consequence of, altered β-oxidation and lipoprotein repackaging as suggested by reduction in breast and thigh muscle lipid content [23]. Broiler bird peripheral fat content is highly associated with dietary fat sources and liver lipid metabolism [24]. Liver and peripheral fat accumulation is also influenced by broiler age and metabolic function at the start of the trials [25]. Although liver fat increased with flaxseed-based diets, body weight was unchanged among the treatments suggesting a larger role of dietary PUFA as a modulator of peripheral fat accumulation. Interestingly, birds fed flaxseed and enzyme had smaller heart mass. The FA composition of this tissue was not measured but warrants further discussion as a measure of avian heart health. Dietary ALA has been shown to increase poultry heart LC n-3 PUFA composition although there were no differences in more functional assays such as ventricle mass ratio [26].

The flaxseed-based diets in our experiment were rich in oleic acid relative to the control, explaining the incorporation of MUFA in hepatic tissue of birds consuming flax or flax+E diets. Presence of MUFA appear essential for the synthesis of hepatic TAG, as observed in mice [27]. MUFA abundance therefore plays a role in TAG packaging and VLDL synthesis in the liver that may be important to understanding commercially-relevant tissue lipid content. Critical to this study is the ratio of essential dietary LC PUFA, ALA:LA, which evidently influenced hepatic FA profiles. Flaxseed supplementation has been previously reported to enrich broiler tissue with ALA [28]; however, our study additionally suggests substantial elongation and desaturation demonstrated by increased LC n-3 FA composition. Flaxseed-fed birds utilized available ALA for elongation and further desaturation to EPA and DHA at the expense of conversion from LA to ARA. The anabolic processes described are likely a result of both dietary influence and substrate availability [28]. Further, multi-carbohydrase enzyme addition enhanced flaxseed hull breakdown to increase substrate availability for LC n-3 PUFA production competitively reducing LC n-6 PUFA synthesis. The results of the current study agree with previous studies that used flaxseed oil to preferentially increase n-3 PUFA in poultry liver tissue at the expense of n-6 PUFA [15,29]. In a follow-up study, carbohydrase enzyme addition in a flaxseed diet enhanced fat digestibility, contributing to availability of beneficial ALA and the differential tissue incorporation of these fats [23].

Phospholipids are important FA cellular reservoirs that modulate metabolic activity as well as secondary signaling molecule production [30]. The FA composition of PC and PE are reflective of both dietary lipid composition and the long chain products of FA synthesis. Phospholipids have a greater unsaturated FA index thus incorporate more LC unsaturated FA (UFA) relative to other lipid classes prompting further investigation into molecular fatty acid species. PL UFA indices can provide a measure of membrane fluidity and dynamics, which may affect signal transduction to alter metabolic pathways [31,32]. We report here that FA synthesis occurs enough so as to differentially enrich PL classes demonstrating both a response to diet and lipid substrate. This response may also be a result of competitive enzymatic activity to enhance LC PUFA synthesis in hepatocytes [33]. The data herein represents a subset of a larger lipidomic evaluation of broiler hepatic tissue. Targeted analyses of the identified lipids may enhance significant alterations in liver tissue by flaxseed and carbohydrase supplementation. Future studies will include more focused approaches to define the role of dietary FA in changing PL FA composition. 

Lipid homeostasis is regulated by a number of key hepatic transcription factors that coordinate the balance of lipid synthesis, oxidation and secretion of FA into circulation for use by peripheral tissue. In the current study, we explored two major hepatic lipid metabolic pathways; biosynthesis regulated by SREBP1 and catabolism regulated by PPARα. *SREBF1* expression was not significantly changed by dietary treatment, although the downstream targets of *de novo* fatty acid synthesis were reduced in flaxseed-fed birds. The n-3 FA pool in the broiler liver reduced the expression of the key *de novo* synthesis genes *FASN* and *ACACA* and the Δ6 desaturase *FADS2*. These results suggest that both dietary composition, specifically ALA, as well as liver total lipids influence mRNA of FA synthesis-associated protein coding genes. The reduction of SREBP1 target gene expression is correlated with reduced whole-body adiposity via repression of lipogenesis and lipid uptake in the broiler liver [34,35]. Lipid accumulation in commercial broiler strains is primarily a consequence of metabolic shift from exponential growth to maintenance and efforts to curb this should be utilized [24]. The avian liver as primary lipid sink is a key target for reducing extra-hepatic tissue fat accumulation [36], with nutritional status closely linked to expression of rate-limiting *ACACA* and *FASN* [37]. Both lipogenic mRNA transcripts were reduced in flax+E birds likely as a consequence of adiposity of the treatment livers [38] and through LC n-3 PUFA modulation of SREBP1 pathways [39]. ALA:LA ratios, however, are considerably more responsible for alterations in transcription of genes coding for desaturase and elongase [40]. *FADS2* is a regulatory checkpoint for conversion of dietary essential FA [41] with activity in monogastric hepatic tissue reduced in response to reduced n-6:n-3 ratio [40]. Other desaturase and elongase genes were not found significantly differentially expressed, suggesting transcriptional regulation of desaturase and elongase genes independent of *SREBF1* status.

PPARα target genes, *ACOX1* and *CPT1A* were up-regulated in hepatic tissue of flax+E birds, an indication of higher FA oxidation in the liver. Dietary PUFA availability via carbohydrase enzyme addition is evidenced to induce β-oxidation through the PPARα pathway [38,42,43]. Although unchanged, PPARα mRNA transcript abundance is responsive to SFA and LC UFA [44,45]. LC n-3 PUFA, synthesized *de novo* and supplied in the diet, produce more robust PPARα downstream responses compared to ALA and LA to reduce fat accumulation and adiposity [46]. PPAR-associated FA activation and ensuing catabolism is similar to SREBP1 pathways in which transcription of necessary genes is mechanistically associated with degree of fatty acid unsaturation as well as total lipid content [38].

Overall, this study demonstrates the effects of dietary n-3 FA supplementation through flaxseed-based diets on broiler liver lipid profile and metabolism. Dietary ALA supplied through flaxseed led to significant enrichment of hepatic PL n-3 molecular species, while addition of enzyme in flaxseed-based diet led to reduction in n-6 species. The effect of enzyme supplementation affected the expression of genes related to lipid metabolism, particularly a decrease expression of genes coding for proteins related to *de novo* FA synthesis and an increase in expression of FA catabolism-related genes. However, the effect of flaxseed without enzyme in the expression of measured genes was minimal.

## Figures and Tables

**Table 1:** Ingredient content, fatty acid composition and calculated nutrient analysis of experimental diets.

Ingredient (g/100 g)	Control	Flax
Corn grain	49.5	43.77
Soybean meal	34.23	31.53
Wheat middlings	8.82	7.72
Corn oil	3.36	-
Canola oil	-	2.92
Limestone	1.98	1.98
Lysine	0.27	0.27
DL-methionine	0.33	0.33
Salt	0.38	0.38
Dicalcium Phosphate	0.61	0.6
Broiler premix ^1^	0.5	0.61
Wheat middlings	8.3	9.1
Flaxseed	-	10
Calculated analysis Metabolizable energy	3186	3189
Crude protein (%)	21.7	22
Fatty acids ^2^ (%)		
14:00	0	0.52
16:00	14.64	7.37
18:00	2.19	2.95
18:01	25.1	34.82
18:2n-6	54.21	27.62
18:3n-3	3.86	26
20:01	0	0.64

^1^ Vitamin-mineral premix supplied per lb feed: vitamin A, 740,000 IU; vitamin D_3_, 440,000 IU; vitamin E, 1200 IU; vitamin B_12_, 1.6 mg; riboflavin, 800 mg; pantothenic acid, 1000 mg; niacin, 6000 mg; menadione, 135 mg; choline, 50,000 mg; thiamine, 275 mg; folic acid, 45 mg; pyridoxine, 180 mg; manganese, 2.5%; zinc, 2.0%; selenium, 120 ppm; copper, 2000 ppm; iodine 1145 ppm; iron 1.8%. ^2^ Analyzed value. Flaxseed was analyzed for crude protein (21.9%), crude fat (41.7%) and gross energy (6174 kcal/kg).

## Supplementary Materials

The following are available online at https://www.mdpi.com/2306-7381/6/1/25/s1, Figure S1: Effect of feeding flax with and without carbohydrase enzyme on hepatic stearoyl-CoA desaturase (SCD) Index of broiler chickens, Table S1: GenBank accession number, hybridization position, sequence, amplicon size, and source of primers for *Gallus gallus domesticus* used to analyze gene expression by qPCR, Table S2: Sequencing results of genes using BLASTN from NCBI (***) against nucleotide collection (nr/nt) with total score, Table S3: Bird body weight at day 21 and 42 of growth with organ yield at day 42, Table S4: Liver total lipids at day 42 of growth, Table S5: Effect of feeding flax with and without carbohydrase enzyme on hepatic fatty acid composition.

## References

[1] Tocher D.R., Bendiksen E.Å., Campbell P.J., Bell J.G. (2008). The role of phospholipids in nutrition and metabolism of teleost fish. Aquaculture.

[2] Calder P.C. (2012). Mechanisms of action of (n-3) fatty acids. J. Nutr..

[3] Wood J.D., Enser M., Fisher A.V., Nute G.R., Sheard P.R., Richardson R.I., Hughes S.I., Whittington F.M. (2008). Fat deposition, fatty acid composition and meat quality: A review. Meat Sci..

[4] Shomonov-Wagner L., Raz A., Leikin-Frenkel A. (2015). Alpha linolenic acid in maternal diet halts the lipid disarray due to saturated fatty acids in the liver of mice offspring at weaning. Lipids Health Dis..

[5] Yang X., Zhuang J., Rao K., Li X., Zhao R. (2010). Effect of early feed restriction on hepatic lipid metabolism and expression of lipogenic genes in broiler chickens. Res. Vet. Sci..

[6] Cohen P., Ntambi J.M., Friedman J.M. (2003). Stearoyl-CoA desaturase-1 and the metabolic syndrome. Curr. Drug Targets Immune Endocr. Metab. Disord..

[7] Jump D.B., Botolin D., Wang Y., Xu J., Christian B., Demeure O. (2005). Fatty Acid Regulation of Hepatic Gene Transcription. J. Nutr..

[8] Rosa F., Osorio J.S., Trevisi E., Yanqui-Rivera F., Estill C.T., Bionaz M. (2017). 2,4-Thiazolidinedione Treatment Improves the Innate Immune Response in Dairy Goats with Induced Subclinical Mastitis. PPAR Res..

[9] Rakhshandehroo M., Knoch B., Müller M., Kersten S. (2010). Peroxisome proliferator-activated receptor alpha target genes. PPAR Res..

[10] Jump D.B. (2008). N-3 polyunsaturated fatty acid regulation of hepatic gene transcription. Curr. Opin. Lipidol..

[11] Ajuyah A.O., Ahn D.U., Hardin R.T., Sim J.S. (1993). Dietary Antioxidants and Storage Affect Chemical Characteristics of ω-3 Fatty Acid Enriched Broiler Chicken Meats. J. Food Sci..

[12] Azcona J.O., Schang M.J., Garcia P.T., Gallinger C., Ayerza R., Coates W. (2008). Omega-3 enriched broiler meat: the influence of dietary α-linolenic-ω-3 fatty acid sources on growth, performance and meat fatty acid composition. Can. J. Anim. Sci..

[13] Bhatty R.S., Cherdkiatgumchai P. (1990). Compositional analysis of laboratory-prepared and commercial samples of linseed meal and of hull isolated from flax. J. Am. Oil Chem. Soc..

[14] Jia W., Slominski B.A. (2010). Means to improve the nutritive value of flaxseed for broiler chickens: the effect of particle size, enzyme addition, and feed pelleting. Poult. Sci..

[15] Apperson K.D., Cherian G. (2017). Effect of whole flax seed and carbohydrase enzymes on gastrointestinal morphology, muscle fatty acids, and production performance in broiler chickens. Poult. Sci..

[16] Folch J., Lees M., Stanley G.H.S. (1957). A simple method for the isolation and purification of total lipides from animal tissues. J. Biol. Chem..

[17] Kansas Lipidomics Research Center. http://www.k-state.edu/lipid/lipidomics/.

[18] Cherian G. (2009). Egg yolk conjugated linoleic acid alters phospholipid molecular species in chick tissues. Eur. J. Lipid Sci. Technol..

[19] Ramakers C., Ruijter J.M., Deprez R.H.L., Moorman A.F. (2003). Assumption-free analysis of quantitative real-time polymerase chain reaction (PCR) data. Neurosci. Lett..

[20] Vandesompele J., De Preter K., Pattyn F., Poppe B., van Roy N., de Paepe A., Speleman F. (2002). Accurate normalization of real-time quantitative RT-PCR data by geometric averaging of multiple internal control genes. Genome Biol..

[21] SAS Institute (2012). SAS User’s Guide. Statistics. Release 9.4.

[22] Smink W., Gerrits W.J., Hovenier R., Geelen M.J., Verstegen M.W., Beynen A.C. (2010). Effect of dietary fat sources on fatty acid deposition and lipid metabolism in broiler chickens. Poult. Sci..

[23] Head B.A., Vercese F., Cherian G. (2017). Total tract lipid digestibility, muscle fatty acids and oxidative stability during storage in broilers fed flax with carbohydrase enzyme. Poult. Sci..

[24] Hermier D. (1997). Lipoprotein metabolism and fattening in poultry. J. Nutr..

[25] Kloareg M., Noblet J., van Milgen J. (2007). Deposition of dietary fatty acids, *de novo* synthesis and anatomical partitioning of fatty acids in finishing pigs. Br. J. Nutr..

[26] Kartikasari L.R., Hughes R., Geier M., Gibson R. (2017). The effect of diet containing high alpha-linolenic acid on omega-3 fatty acids and health status of the heart in broilers. Bul. Peternak..

[27] Miyazaki M., Kim Y.C., Ntambi J.M. (2001). A lipogenic diet in mice with a disruption of the stearoyl-CoA desaturase 1 gene reveals a stringent requirement of endogenous monounsaturated fatty acids for triglyceride synthesis. J. Lipid Res..

[28] Betti M., Perez T.I., Zuidhof M.J., Renema R.A. (2009). Omega-3-enriched broiler meat: 3. Fatty acid distribution between triacylglycerol and phospholipid classes. Poult. Sci..

[29] Kartikasari L.R., Hughes R.J., Geier M.S., Makrides M., Gibson R.A. (2012). Dietary alpha-linolenic acid enhances omega-3 long chain polyunsaturated fatty acid levels in chicken tissues. Prostaglandins Leukot Essent Fat. Acids.

[30] Sugiura Y., Konishi Y., Zaima N., Kajihara S., Nakanishi H., Taguchi R., Setou M. (2009). Visualization of the cell-selective distribution of PUFA-containing phosphatidylcholines in mouse brain by imaging mass spectrometry. J. Lipid Res..

[31] Hulbert A.J., Else P.L. (1999). Membranes as possible pacemakers of metabolism. J. Biol..

[32] Clarke S.D., Jump D.B. (1996). Polyunsaturated fatty acid regulation of hepatic gene transcription. Lipids.

[33] Bezard J., Blond J.P., Bernard A., Clouet P. (1994). The metabolism and availability of essential fatty acids in animal and human tissues. Reprod. Nutr. Dev..

[34] Assaf S., Lagarrigue S., Daval S., Sansom M., Leclercq B., Michel J., Pitel F., Alizadeh M., Vignal A., Douaire M. (2004). Genetic linkage and expression analysis of SREBP and lipogenic genes in fat and lean chicken. Comp. Biochem. Physiol. Part B Biochem. Mol. Biol..

[35] Zhang L., Li C., Wang F., Zhou S., Shangguan M., Xue L., Zhang B., Ding F., Hui D., Liang A., He D. (2015). Treatment with PPARalpha agonist clofibrate inhibits the transcription and activation of SREBPs and reduces triglyceride and cholesterol levels in liver of broiler chickens. PPAR Res..

[36] Ding F., Pan Z., Kou J., Li L., Xia L., Hu S., Liu H., Wang J. (2012). *De novo* lipogenesis in the liver and adipose tissues of ducks during early growth stages after hatching. Comp. Biochem. Physiol. Part B Biochem. Mol. Biol..

[37] Wang P.H., Ko Y.H., Chin H.J., Hsu C., Ding S.T., Chen C.Y. (2009). The effect of feed restriction on expression of hepatic lipogenic genes in broiler chickens and the function of SREBP1. Comp. Biochem. Physiol. Part B Biochem. Mol. Biol..

[38] Sanz M., Lopez-Bote C.J., Menoyo D., Bautista J.M. (2000). Abdominal fat deposition and fatty acid synthesis are lower and beta-oxidation is higher in broiler chickens fed diets containing unsaturated rather than saturated fat. J. Nutr..

[39] Duran-Montgé P., Theil P.K., Lauridsen C., Esteve-Garcia E. (2009). Dietary fat source affects metabolism of fatty acids in pigs as evaluated by altered expression of lipogenic genes in liver and adipose tissues. Animal.

[40] Jing M., Gakhar N., Gibson R.A., House J.D. (2013). Dietary and ontogenic regulation of fatty acid desaturase and elongase expression in broiler chickens. Prostaglandins Leukot Essent Fat. Acids.

[41] Campioli E., Rustichelli C., Avallone R. (2012). n-3 Dietary supplementation and lipid metabolism: Differences between vegetable- and fish-derived oils. J. Funct. Foods.

[42] Ortiz L.T., Rebole A., Alzueta C., Rodriguez M.L., Trevino J. (2001). Metabolisable energy value and digestibility of fat and fatty acids in linseed determined with growing broiler chickens. Br. Poult. Sci..

[43] Newman R.E., Bryden W.L., Fleck E., Ashes J.R., Buttemer W.A., Storlien L.H., Downing J.A. (2002). Dietary *n*-3 and *n*-6 fatty acids alter avian metabolism: metabolism and abdominal fat deposition. Br. J. Nutr..

[44] Varga T., Czimmerer Z., Nagy L. (2011). PPARs are a unique set of fatty acid regulated transcription factors controlling both lipid metabolism and inflammation. Biochim. Biophys. Acta.

[45] Vallim T., Salter A.M. (2010). Regulation of hepatic gene expression by saturated fatty acids. Prostaglandins Leukot Essent Fat. Acids.

[46] Schmitz G., Ecker J. (2008). The opposing effects of *n*−3 and *n*−6 fatty acids. Prog. Lipid Res..

