# Supplementary material for: Flaxseed and Carbohydrase Enzyme Supplementation Alters Hepatic n-3 Polyunsaturated Fatty Acid Molecular Species and Expression of Genes Associated with Lipid Metabolism in Broiler Chickens
Source: Vet Sci. 2019 Mar 8;6(1):25. doi: 10.3390/vetsci6010025 (PMC6465989; doi:10.3390/vetsci6010025)
Supplement: Supplementary file 1 [file vetsci-06-00025-s001.pdf]

Table S1. GenBank accession number, hybridization position, sequence, amplicon size, and source of primers for *Gallus gallus domesticus* used to analyze gene expression by qPCR.

Accession #	Gene	Primers*	Primers (5'-3')**	bp***	Source
NM_001001464	PPARA	F 1553	GTGGAGATCGTCCTGGTCTTG	100	This manuscript
		R 1652	TCAGGATGGTTGGTTTGCAA		
NM_001006205.1	ACOX1	F 599	GCCAGGTGGACTTGGAAAGA	107	This manuscript
		R 705	GCTGCCGTATAGGAACAATGAAG		
NM_001012578.1	ACSL1	F 1839	GAGAGCTTGCAGGCCTTCTTAC	103	This manuscript
		R 1941	ATAGCTTCTCATATGTGCCTTCAAATC		
NM_001012898	CPT1A	F 1807	GCCCTGATGCCTTCATTCAA	60	This manuscript
		R 1866	ATTTTCCCATGTCTCGGTAGTGA		
NM_001044633.1	ApoB100	F 1511	TCACACTTCGGGCTATTGGAA	101	This manuscript
		R 1611	TGATGCAGCTTGATTTCTGATACA		
NM_204192.3	FABP1	F 271	AGAAGGCCAAGTGTATTGTTAACATG	100	This manuscript
		R 370	GTGATGGTGTCTCCGTTGAGTTC		
XM_424010	SLC27A2	F 1174	CAACGTGTTCCTCAAGGCTTTTA	100	This manuscript
		R 1273	GACGCGGATGCAGAATCC		
XM_419374	DGAT2	F 928	ATTGGCTTTGCTCCATGCAT	100	This manuscript
		R 1027	CGCCCACAACAGTGGTGAT		
NM_204126.2	SREBF-1	F 1058	GCGCTACCGCTCATCCAT	93	This manuscript
		R 1150	CTCAGGATCGCCGACTTGTT		
NM_205155	FASN	F 6211	TCTGCCATGGAGCGTATCTG	136	This manuscript
		R 6346	GAACGGTTCCCCCAATCAC		
NM_001109784.2	MTTP	F 2049	CTGGCCAGGTGGTGATTGA	100	This manuscript
		R 2148	GCTGACATGCCAGCAAAGG		
NM_205505	ACCA	F 5308	GATGACCCATACAAAGGATACAAGTA	123	This manuscript
		R 5430	TATCTTATACCTGGACTCTCCGTTGTC		
XM_004941487.1	FADS1	F 859	CCCAACTGCTTCCGAAAGG	92	This manuscript
		R 950	TTTTGTTCGCCAAGCTCTACAG		
NM_001160428	FADS2	F 797	CACTTCCAACATCACGCTAAGC	102	This manuscript
		R 898	GCCGTACTCAATAGGCTGACTTTC		
NM_204890.1	SCD1	F 383	TGGCATGGGCCATTCTGT	61	This manuscript
		R 443	CGGTGAGATCCAGCTGTTATCC		
NM_001031539.1	Elovl6	F 351	GCCTTGCTGTCTTCAGTATATTCG	100	This manuscript
		R 450	CTCTGGTCACACACTGACTGCTT		
NM_204305.1	GAPDH	F 194	AATATGATTCTACACACGGACACTTCA	100	This manuscript
		R 293	TCACGCTCCTGGAAGATAGTGA		
BX936152	MRPL39	F 69	AATGGCGCAGGTTCAACTCT	100	This manuscript
		R 168	TGCCTCTCCTTCTCTTTGGTAAA		
NM_001277758.1	MRPS9	F 907	AGGCTGGAGCGATACGTTTG	90	This manuscript
		R 996	GCAGCCCAGCTTGTCTCATAA		
XM_004945286.1	RPS15A	F 200	AGCATGGTTACATTGGTGAATTTG	80	This manuscript
		R 279	GAGTCTGCCTGTGAGATTGACAA		

* Primer direction (F—forward; R—reverse) and hybridization position on the sequence. ** Exon-exon junctions are underlined. *** Amplicon size in base pairs (bp).

Table S2. Sequencing results of genes using BLASTN from NCBI (***) against nucleotide collection (nr / nt) with total score.

Gene	Best hit in NCBI	Score
PPARA	Gallus gallus peroxisome proliferator-activated receptor alpha (PPARA)	39.1
ACOX1	Gallus gallus acyl-CoA oxidase 1, palmitoyl (ACOX1)	31.9
ACSL1	Gallus gallus acyl-CoA synthetase long-chain family member 1 (ACSL1)	50
CPT1	Gallus gallus carnitine palmitoyltransferase 1A (liver) (CPT1A)	42.8
ApoB100	Gallus gallus apolipoprotein B (APOB)	44.6
FABP1	Gallus gallus fatty acid binding protein 1, liver (FABP1)	48.2
SLC27A2	PREDICTED: Gallus gallus solute carrier family 27 (fatty acid transporter), member 2 (SLC27A[illegible]	42.8
DGAT2	PREDICTED: Gallus gallus diacylglycerol O-acyltransferase homolog 2 (mouse) (DGAT2)	37.4
SREBF-1	Gallus gallus sterol regulatory element binding transcription factor 1 (SREBF1)	37.4
FASN	Gallus gallus fatty acid synthase (FASN)	37.4
MTTP	Gallus gallus microsomal triglyceride transfer protein (MTTP)	35.6
ACCA	Gallus gallus acetyl-CoA carboxylase alpha (ACACA)	51.8
FADS1	Gallus gallus fatty acid desaturase 1 (FADS1)	41
FADS2	Gallus gallus fatty acid desaturase 2 (FADS2)	44.6
SCD1	Gallus gallus stearoyl-CoA desaturase (delta-9-desaturase) (SCD)	41
Elovl6	Gallus gallus elongation of very long chain fatty acids family member protein 6 (ELOVL6)	44.6
GAPDH	Gallus gallus glyceraldehyde-3-phosphate dehydrogenase (GAPDH)	50
MRPL39	PREDICTED: Gallus gallus mitochondrial ribosomal protein L39 (MRPL39), transcript variant X[illegible]	42.8
MRPS9	Gallus gallus mitochondrial ribosomal protein S9 (MRPS9), transcript variant 1	39.2
RPS15A	PREDICTED: Gallus gallus ribosomal protein S15a (RPS15A), transcript variant X4	44.6

Tabel S3. Bird body weight at day 21 and 42 of growth with organ yield at day 42.

Growth parameters	Dietary Treatment[1]			Pooled SEM	P value	
	Control	Flax	Flax+E		Control vs. Flax	Flax vs. Enzyme
Initial body weight (g)	79.08	79.05	79.15	0.80	0.992	0.971
Day 21 weight (g)	467.17	457.19	458.35	2.46	0.659	0.962
Final body weight (kg)	1.68	1.59	1.70	0.19	0.776	0.432

Organ yield (% of BW)	Dietary Treatment[1]			Pooled SEM	P value	
	Control	Flax	Flax+E		Control vs. Flax	Flax vs. Enzyme
Breast muscle	25.03	24.49	25.54	0.55	0.987	0.404
Thigh muscle	8.92	9.52	9.53	0.36	0.187	0.988
Heart	0.68^{a}	0.67^{a}	0.57^{b}	0.11	0.126	0.033

[1] Control, Flax, Flax+E represent corn-soybean meal basal diet (Control), and basal diet with 10% whole flaxseed (Flax) plus 0.05% enzyme (Flax+E). $n = 8$. ^{a,b} Means within a row with no common superscript differ when $p < 0.05$. BW = Body weight.

Table S4. Liver total lipids at day 42 of growth.

Total Lipids	Dietary Treatments[1]			Pooled SEM	P value	
	Control	Flax	Flax+E		Control vs. Flax	Flax vs. Enzyme
Liver (mg/g tissue)	3.31^{b}	3.59^{a}	3.77^{a}	0.22	0.044	0.364

[1] Control, Flax, Flax+E represent corn-soybean meal basal diet (Control), and basal diet with 10% whole flaxseed (Flax) plus 0.05% enzyme (Flax+E). $n = 8$. ^{a,b} Means within a row with no common superscript differ when $p < 0.05$.

Table S5. Effect of feeding flax with and without carbohydrase enzyme on hepatic fatty acid composition.

Fatty Acid (g/100 g FAME)	Dietary Treatments[1]			Pooled SEM	p-value	
	Control	Flax	Flax+E		Control vs. flax	Flax vs. enzyme
14:0	0.32[ab]	0.25[b]	0.39[a]	0.13	0.9281	0.0492
16:0	16.59	15.46	16.37	1.77	0.413	0.3189
16:1	0.72[b]	1.12[ab]	1.55[a]	0.43	0.0048	0.057
17:0	0.46	0.58	0.47	0.25	0.5894	0.4096
18:0	23.22[a]	22.60[a]	19.70[b]	2.03	0.0353	0.0097
18:1	9.62[c]	14.26[b]	18.56[a]	3.55	0.0004	0.0251
18:2 n-6	23.07[a]	20.71[b]	20.35[b]	1.75	0.0044	0.6849
18:3 n-3	0.32[b]	2.20[a]	2.91[a]	1.01	<0.0001	0.1745
20:1	0.33	0.62	0.67	0.33	0.0495	0.7576
20:3 n-6	1.30[a]	0.73[b]	0.66[b]	0.23	<0.0001	0.5709
20:3 n-3	1.02	1.31	1.10	0.31	0.1934	0.1998
20:4 n-6	16.68[a]	10.78[b]	9.01[c]	1.48	<0.0001	0.027
20:5 n-3	0.06[b]	2.48[a]	1.78[a]	1.08	0.0004	0.2059
22:4 n-6	1.37[a]	0.59[b]	0.48[b]	0.21	<0.0001	0.3128
22:5 n-6	2.11[a]	0.25[b]	0.33[b]	0.27	<0.0001	0.5509
22:5 n-3	0.50[b]	1.30[a]	1.29[a]	0.29	<0.0001	0.9449
22:6 n-3	2.30[b]	4.79[a]	4.39[a]	0.94	<0.0001	0.4098
Total SFA	40.59[a]	38.89[ab]	36.94[b]	2.11	0.0109	0.0786
Total MUFA	10.67[c]	16.00[b]	20.77[a]	3.99	0.0004	0.0266
Total n-6 FA	43.23[a]	32.32[b]	30.17[b]	2.19	<0.0001	0.0642
Total n-3 FA	5.51[b]	12.80[a]	12.13[a]	2.46	<0.0001	0.5896
Total LC n-6	20.16[a]	11.61[b]	9.82[c]	1.70	<0.0001	0.0471
Total LC n-3	5.19[b]	10.61[a]	9.22[a]	1.92	<0.0001	0.1635

[1] Control, Flax, Flax+E represent corn-soybean meal basal diet (Control), and basal diet with 10% whole flaxseed (Flax) plus 0.05% enzyme (Flax+E). $n = 8$. [a–c] Means within a row with no common superscript differ when $p < 0.05$. SEM = Standard error of the mean.

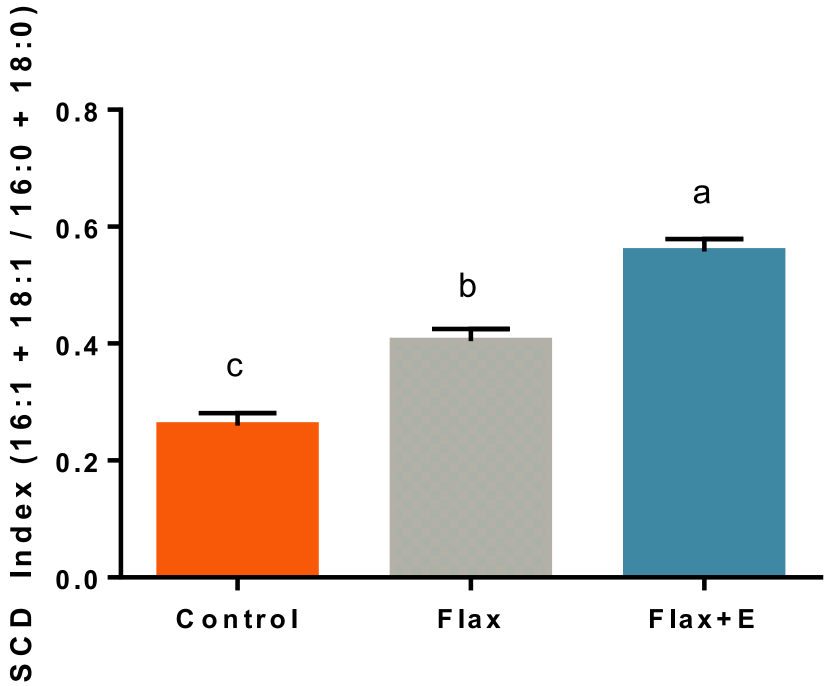

Figure S1. Effect of feeding flax with and without carbohydrase enzyme on hepatic Stearoyl-CoA Desaturase (SCD) Index of broiler chickens.

[1] Control, Flax, Flax+E represent corn-soybean meal basal diet (Control), and basal diet with 10% whole flaxseed (Flax) plus 0.05% enzyme (Flax+E). [a–c] Means with no common superscript differ when $p < 0.05$. $n = 8$.
